# Resumptive *Streptococcus mutans* Persisters Induced From Dimethylaminododecyl Methacrylate Elevated the Cariogenic Virulence by Up-Regulating the Quorum-Sensing and VicRK Pathway Genes

**DOI:** 10.3389/fmicb.2019.03102

**Published:** 2020-01-21

**Authors:** Junzhuo Lu, Lei Cheng, Yuyao Huang, Yaling Jiang, Chun-Hung Chu, Xian Peng, Mingyun Li, Hockin H. K. Xu, Xuedong Zhou, Biao Ren

**Affiliations:** ^1^State Key Laboratory of Oral Diseases, National Clinical Research Center for Oral Diseases, West China Hospital of Stomatology, Sichuan University, Chengdu, China; ^2^Faculty of Dentistry, The University of Hong Kong, Hong Kong, China; ^3^Department of Advanced Oral Sciences and Therapeutics, University of Maryland School of Dentistry, Baltimore, MD, United States

**Keywords:** dimethylaminododecyl methacrylate, anti-bacterial material, drug tolerance, persister cells, cariogenic virulence

## Abstract

Bacterial persistence has become a worldwide health problem due to its ability to cause the recalcitrance and relapse of infections. The existence of bacterial persistence and their possible mechanisms have been widely reported. However, the following regrowth of persister cells is not clear although the awakening of dormant surviving persisters is the key to reinitialize bacterial infection. In this study, we investigated the growth character and cariogenic virulence during the recovery of *Streptococcus mutans* drug-tolerant persister cells induced by a novel quaternary ammonium: dimethylaminododecyl methacrylate (DMADDM). A remarkable lag phase was observed in *S. mutans* persisters when regrew at the first 24 h compared to normal cells. During the entire recovery state, persisters are metabolically active to increase the production of both water-soluble and water-insoluble glucan. The shortage of cell number in persisters resulted in the decrease of lactic acid production, but persisters gradually recovered the normal acid production ability after 72 h. The up-regulated expression of *gtf* and *vicR* was in line with comDE circuit and consistent with the virulence change during the regrowth stage. Our findings proved that lethal dosages of DMADDM induced drug-tolerant *S. mutans* persisters in biofilm, which had a prolonged lag phase and elevated cariogenic virulence during regrowth. The recovery and elevated virulence of persisters were regulated by quorum-sensing and VicRK pathway. This alarmed the elevated cariogenicity of persisters and highlighted the critical requirement for the drug-tolerance evaluation when developing new oral antimicrobial agents. To the best of our knowledge, we characterized the regrowth and cariogenic virulence variation of *S. mutans* persisters induced by quaternary ammonium for the first time. Our findings suggest that *S. mutans* persisters with the elevated cariogenic virulence during their regrowth stage highlighted the need of new strategy to overcome bacterial persistence. Meanwhile, the prolonged lag phase and the involvement of quorum-sensing system in the regrowth of *S. mutans* persisters may provide the potential targets.

## Introduction

The use of antibiotics/antimicrobial agents has saved millions of lives over the years; however, the rapid growth of antimicrobial resistance (AMR) has become an urgent threat to global health. AMR could cause >2 million infections with 23,000 and over 38,000 deaths annually in the United States and Asia, respectively (Holmstrup and Klausen, 2016). Bacterial survival under antibiotic stress is usually characterized by two major factors – resistance and tolerance (Brauner et al., 2016). Resistance describes the inherited ability of bacteria to grow at high concentrations of an antibiotic (McKeegan et al., 2002). Tolerance is the ability of the whole microbial population to survive from the lethal concentrations of antibiotics without a change in the minimum inhibitory concentration (MIC) by slowing down the essential bacterial process (Horne and Tomasz, 1977). Another mode of AMR pattern named “persistence” is similar with tolerance as persister can also survive from the lethal concentrations of antibiotics without the change at MIC. However, persistence is the ability of a subpopulation surviving from the exposure of high concentrations of antibiotics (Gefen and Balaban, 2009). Persistence is also unlike the resistance bacteria which usually acquire the heritable gene mutations, while persisters are genetically identical to regular cells in genome but show a distinct antibiotic tolerant phenotype through a state of dormancy (Cohen et al., 2013).

In recent years, bacterial persistence has become a worldwide health problem due to its ability to cause the recalcitrance and relapse of infections (Balaban et al., 2013). Persister cells have also been linked to biofilm multi-drug tolerance and the increase of antibiotic resistance during treatment (Lewis, 2007). The possible mechanisms of persistence in different bacterial pathogens have been reported during the recent decades, including the well-established gene pairs known as toxin–antitoxin (TA) systems, a gene loci persistent cells display increased expression as is the case for *Escherichia coli hipA* mutant, and the enhanced efflux activity in that persisters accumulate fewer antibiotics as is the case for high expression of *tolC* in *E. coli*, and by “hiding” inside macrophages within granulomas as is the case for *M. tuberculosis* and *S. typhi*, and antigenic variation in *Borrelia burgdorferi* (Monack et al., 2004; Palmer et al., 2009; Helaine and Kugelberg, 2014; Page and Peti, 2016; Pu et al., 2016). However, the following regrowth of persister cells is not clear although the awakening of dormant surviving persisters is the key to reinitialize bacterial infection (Mina and Marques, 2016). Understanding the recovery characters of the persisters for the dormant stage is important to develop effective methods to eradicate persisters and further to reduce the post infections (Lewis and Shan, 2016).

The oral environment is exposed to a wide range of environmental conditions including fluctuations of pH and various antimicrobials. However, only a few oral microbes were reported about their persistent abilities under different antimicrobials. The oral fungus *Candida albicans* can form persisters treated with chlorhexidine and the bacterium *Streptococcus mutans*, the primary etiological agents of dental caries, also formed persisters when challenged with ciprofloxacin (Lafleur et al., 2010; Leung et al., 2015). It was reported that quorum-sensing system, the CSP-ComDE regulatory circuit, was associated with the formation of *S. mutans* persisters when treated by ciprofloxacin. The stress-inducible persistence phenotype was abolished in the Δ*comE* mutant, which is unable to respond to the CSP signaling pheromone (Leung and Levesque, 2012).

Quaternary ammonium salts (QASs) can incorporate into many dental materials to combat oral bacterial species (Imazato, 2009; Cheng et al., 2017) by damaging the cell membrane (Beyth et al., 2006). A novel quaternary ammonium monomer, dimethylaminododecyl methacrylate (DMADDM) was proved to be a potential anti-caries dental material due to its long-lasting and remarkable antibacterial effects and well biocompatibility *in vitro* and *in vivo* (Cheng et al., 2013; Mei et al., 2013; Wang et al., 2016; Zhang et al., 2016). We identified that lethal concentration of DMADDM can induce the formation of *S. mutans* persisters by decreasing the metabolism for the first time (Jiang et al., 2017). The induction of *S. mutans* persistence by DMADDM highlighted the critical evaluation of drug resistance/tolerance when developing antibacterial dental materials. However, the recovery and virulence change of *S. mutans* persisters awakening from the persistent status has not been investigated yet. In order to enrich the characteristics of *S. mutans* persisters and highlight the necessity of drug tolerance evaluation for antimicrobial dental materials, we reported the landscape of the regrowth progress the dynamic virulent variation of *S. mutans* persisters induced by DMADDM during the recovery stage in this study for the first time.

## Materials and Methods

### Synthesis of DMADDM

The antibacterial quaternary ammonium DMADDM used in this study was synthesized via a modified Menschutkin reaction as elaborately described previously (Cheng et al., 2013). Briefly, 2.13 g of the tertiary amine 1-(dimethylamino) dodecane (DMAD, Tokyo Chemical Industry, Tokyo, Japan), 1.91 g of organo halide BEMA (Monomer–Polymer and Dajac Labs, Trevose, PA, United States), and 3 g of ethanol (Kelong Chemical, Chengdu, China) were added to a 20-ml scintillation vial with a magnetic stir bar. The vial was capped and stirred at 70°C for 24 h. After the reaction was completed, the ethanol solvent was removed via evaporation. The yielded DMADDM is a clear, colorless, and viscous liquid. To characterize the successful synthesis of DMADDM, Fourier transform infrared (FTIR) spectroscopy (Nicolet 6700, Thermo Scientific, Waltham, MA, United States) spectra were collected between two KBr windows in the 4000–400 cm^–1^ region, and ^1^H NMR spectra (GSX 270, JEOL) were taken in deuterated chloroform at a concentration of about 3% for assigning peaks to the alkyl group of the monomer. The reactions and products of DMADDM were all verified in preliminary studies (Liang et al., 2018).

### Bacterial Strain and Growth Media

*Streptococcus mutans* strain UA159 (ATCC 700610) provided by the State Key Laboratory of Oral Diseases (Sichuan University, Chengdu, China) was cultured in brain–heart infusion broth (BHI; BD, Franklin Lakes, NJ, United States) medium at 37°C anaerobically (90% N_2_, 5% CO_2_, and 5% H_2_). The biofilm was initiated by adding 1% sucrose in 1 ml BHI medium in 48-well plate (Corning, NY, United States). The medium was changed with fresh medium every 24 h.

### The Formation of *S. mutans* Persister Cells

We first determined the MIC of DMADDM against *S. mutans* strain UA159 via serial microdilution assays as described in previous study (Cheng et al., 2013). Then different concentrations of DMADDM above the MIC were employed to induce the *S. mutans* persisters. The persistent cells were then evaluated in time-dependence killing curve as described in previous work (Jiang et al., 2017). In brief, aliquots of overnight culture of *S. mutans* were diluted in BHI broth to obtain the final concentration of 1 × 10^6^ CFU/ml. Biofilm growth was initiated by inoculating 1 ml of *S. mutans* BHIS suspension into each well of a 48-well plate and incubating it for 24 h to develop a biofilm on the bottom of the plate. After 24 h, biofilms were rinsed three times with sterile phosphate-buffered saline (PBS) to remove the planktonic and loose binding cells. Fresh culture medium containing either 100 (10× MIC) or 120 μg/ml (12× MIC) of DMADDM was added and treated for another 24–72 h to induce persister cells. Samples were collected at different indicated time points. The collected samples were serially diluted and then plated on BHI agar plates to recover the viable cells. Colonies were counted after 48 h of incubation at 37°C. To testify whether DMADDM truly increases the formation of *S. mutans* persisters in biofilms, we exposed cells with a range of DMADDM concentrations above MIC. In brief, *S. mutans* at a concentration of 10^6^ CFU/ml were placed into each well of 48-well plates and incubated overnight at 37°C with 5% CO_2_ for 24 h to form mature biofilms. Then biofilms were rinsed three times with sterile PBS to remove the planktonic and loose binding cells. Fresh culture medium containing 0–200 μg/ml DMADDM was added to test surviving level of persister cells. Samples at 24 h were collected for colony counting. To test the heritability of AMR, the surviving colonies were re-grown in fresh BHI broth for 16 h at 37°C for inoculation preparation. Then the biofilm was initiated, followed with the treatment of DMADDM and collected for another colony counting as described above. This procedure was repeated for three consecutive cycles. The MIC of the surviving population in each cycle was tested as described above.

### Growth Resumption

The *S. mutans* persisters in the biofilm was induced as described. After 24 h, the supernatant was removed, then the biofilm was rinsed twice with sterile PBS to remove the residual DMADDM. Fresh BHI broth supplemented with 1% sucrose was added to initiate the growth resumption of the surviving persister cells. The regrown biofilm was incubated for another 24–96 h. Bacterial culture medium was changed every 24 h. Samples were collected and tested at indicated time. The untreated seed *S. mutans* cells (10^5^ CFU/ml, equal to the amount of 100 μg/ml DMADDM induced persisters in the biofilm) were also incubated for 24–96 h as control. The schematic illustration of experimental route was shown in Supplementary Figure S1.

### Biofilm Imaging

After 24, 48, 72, and 96 h, the biofilms on the disks were washed with PBS, and then stained using the BacLight live/dead bacterial viability kit (Molecular Probes, Invitrogen). Live bacteria were stained with SYTO9 to produce green fluorescence and dead bacteria were stained with propidium iodide to produce red fluorescence. The labeled biofilms were imaged with a confocal laser scanning microscope (Leica, Wetzlar, Germany) equipped with a 20× objective lens. The three-dimensional reconstruction and quantification of live/dead fluorescence intensity of the biofilms were performed with Imaris 7.0.0 (Bitplane, Zürich, Switzerland) and Image-Pro Plus (Media Cybernetics, Silver Spring, MD, United States), respectively (Zhang et al., 2015).

### Lactic Acid Measurement and pH Measurement

Lactate concentrations of the biofilm were determined using an enzymatic (lactate dehydrogenase) method (van Loveren et al., 2000). Briefly, the bacteria and culture medium of biofilms were collected and centrifugated at 4000 rpm for 10 min at 4°C. The supernatants were decanted to measure lactate concentrations according to the manuscript of the Lactate Assay Kit (MAK064, Sigma, United States). The standard curves were prepared with a lactic acid standard (Supelco, Bellefonte, PA, United States). The absorbance at 570 nm was recorded using a microplate reader (Gene, Hong Kong) and lactate concentrations were then calculated according to the standard curves. For pH measurement, 1 ml supernatant of the biofilms was measured by Orion Dual Star, pH/ISE Benchtop (Thermo Scientific, Waltham, United States). Both regrowth persisters and untreated seed biofilms were collected at 0, 24, 48, 72, and 96 h for the measurement.

### Polysaccharide Measurement

The biofilm polysaccharides were determined by an anthrone sulfuric acid colorimetric assay (Koo et al., 2003). The standard curves were drawn by measuring different concentration gradient of glucan. Briefly, the bacterial biofilms were collected at 4000 rpm for 30 min at 4°C. The supernatant mixed with 95% ethanol was maintained 4°C for 24 h, and the water-soluble glucans were obtained by centrifugation. The water-soluble glucans and the precipitate water-insoluble glucans were washed twice with 4 ml of 0.4 M NaOH. After centrifugation, 200 μl of supernatant mixed with 600 μl of anthrone–sulfuric acid reagent was maintained 95°C for 6 min, then cooled down to room temperature and read its absorbance at 625 nm. Both persister regrowth and untreated seed control biofilms were withdrawn at 24, 48, 72, and 96 h for the measurement. The pre-accumulation of water-insoluble glucans before the regrowth of persisters in the biofilm was excluded by subtracting the quantity of water-insoluble glucans at 0 h of regrowth when DMADDM was removed.

### Extraction of RNA and Real-Time RT-PCR

Quantitative RT-PCR was used to quantify expression of selected genes (gene names and descriptions are shown in Supplementary Tables S1, S2), with 16S rRNA as an internal control. The RNA extraction and reverse transcriptase PCR (RT-PCR) conditions and primers were described previously (Peng et al., 2016). Briefly, resumptive persister and untreated seed biofilm cells were harvested at indicated time points by centrifugation. Total RNA was isolated from cells using the TRizol regent (Invitrogen). cDNAs were synthesized using PrimeScript^TM^ RT reagent kit with gDNA Eraser (RR047A; Takara Bio, Shiga, Japan). Tested genes and specific primers were listed in Supplementary Table S1. Real-time PCRs were performed using SYBR^®^ Premix Ex Taq^TM^ (Tli RNaseH Plus) from Takara Bio, Shiga, Japan. Threshold cycle values (CT) were determined, and the data were analyzed by BIO-RAD CFX MANAGER software (version 2.0) using the 2^–ΔΔ*CT*^ method.

### Statistical Analysis

Each of the experiments was conducted using triplicate wells and done at least three times. Student’s *t*-test was used to compare the data of two groups, and data were considered significantly different if the two-tailed *P*-value was <0.05. Statistical analysis was performed with the SPSS software, version 23.0 (IBM SPSS Inc., Chicago, IL, United States).

## Results

### DMADDM Induced Non-heritable Tolerant Persisters in *S. mutans* Biofilm

**FIGURE 1:**
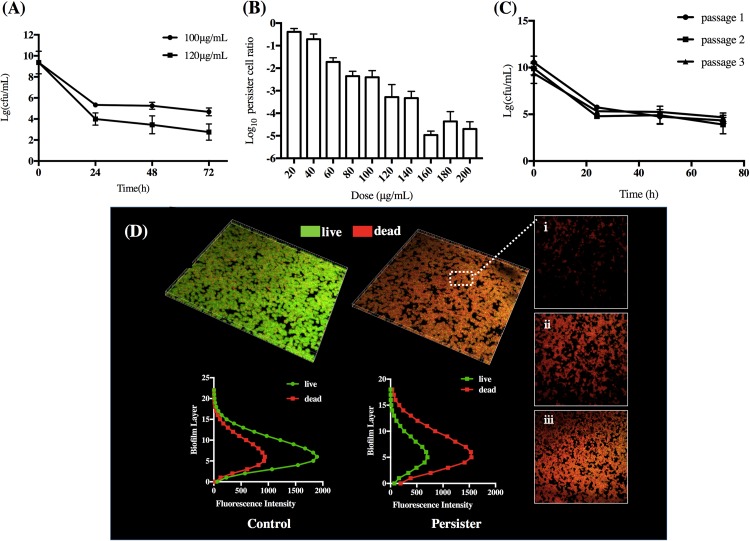
**(A)** Time-dependence biphasic killing curve. **(B)** Persister levels after 24 h under a range of concentrations of DMADDM challenge. **(C)** The biphasic killing curve of three consecutive cycles. **(D)** The CLSM imaging and fluorescence distribution of *S. mutans* persisters and untreated normal biofilm. The pictures on the right panel displayed high magnification imaging of (i) top, (ii) middle, and (iii) bottom layer of persister biofilm (live bacteria, stained green; dead bacteria, stained red). The data shown in panels **A**–**C** are the means of three independent experiments. The error bars indicate the standard deviations of the means.

When *S. mutans* biofilms were exposed to DMADDM, there was a rapid decrease of bacterial CFU within first 24 h and a subsequent plateau phase in the following 72 h, indicating the stable persister cells in the treated biofilm. *S. mutans* exposed to 10× MIC and 12× MIC concentration of DMADDM significantly decreased from 10^8^ to about 10^5^ and 10^4^ CFU/mL, respectively (Figure 1A). To testify whether the *S. mutans* persisters in biofilms were induced by exposure to DMADDM rather than pre-existing, the cells were exposed with a range of DMADDM concentrations above MIC. The persister level with exposure to 100 μg/ml was about 100- to 700-fold higher in the cultures treated with high concentrations of DMADDM (120–200 μg/ml), while persister level was reduced 10- to 800-fold compared to cultures treated with low concentrations of DMADDM (20–80 μg/ml). Increased concentration of DMADDM led to decreased persister level (Figure 1B). This indicated that persister cells in the biofilm were induced by DMADDM rather than pre-existing. As if they were pre-existing, the bulk would not be killed by any bactericidal concentration of DMADDM and keep the same level of persister population. The biofilm structure of the surviving pesisters was further observed by CLSM imaging. Live bacteria were stained green, and dead bacteria were stained red. Persister biofilm displayed mainly red staining, indicating that majority of the population were killed by DMADDM (Figure 1D). CLSM imaging from bottom to the top of the persister biofilm proved the undifferentiated killing effect of DMADDM to the whole biofilm. The fluorescence intensity at variety height of the biofilm showed that live/dead bacteria distribution was average in each layer which was similar with the seed control group (Figure 1D), indicating the survival of persisters was not due to the lack of penetration of antimicrobial agent. Then we re-cultivated the persisters to a new population followed by the exposure to DMADDM, the cells were also sensitive to DMADDM and presented the similar biphasic-killing curves irrespective of the number of passages (Figure 1C). MICs of initial biofilm population and the surviving subpopulation persisters remained unchanged in three tested cycles (Supplementary Table S3), indicating the formation of *S. mutans* persisters in biofilm when treating with lethal concentrations of DMADDM.

### The *S. mutans* Persister Cells Formed an Actively Growing Biofilm With Extended Lag Time at Early Stage of Regrowth

**FIGURE 2:**
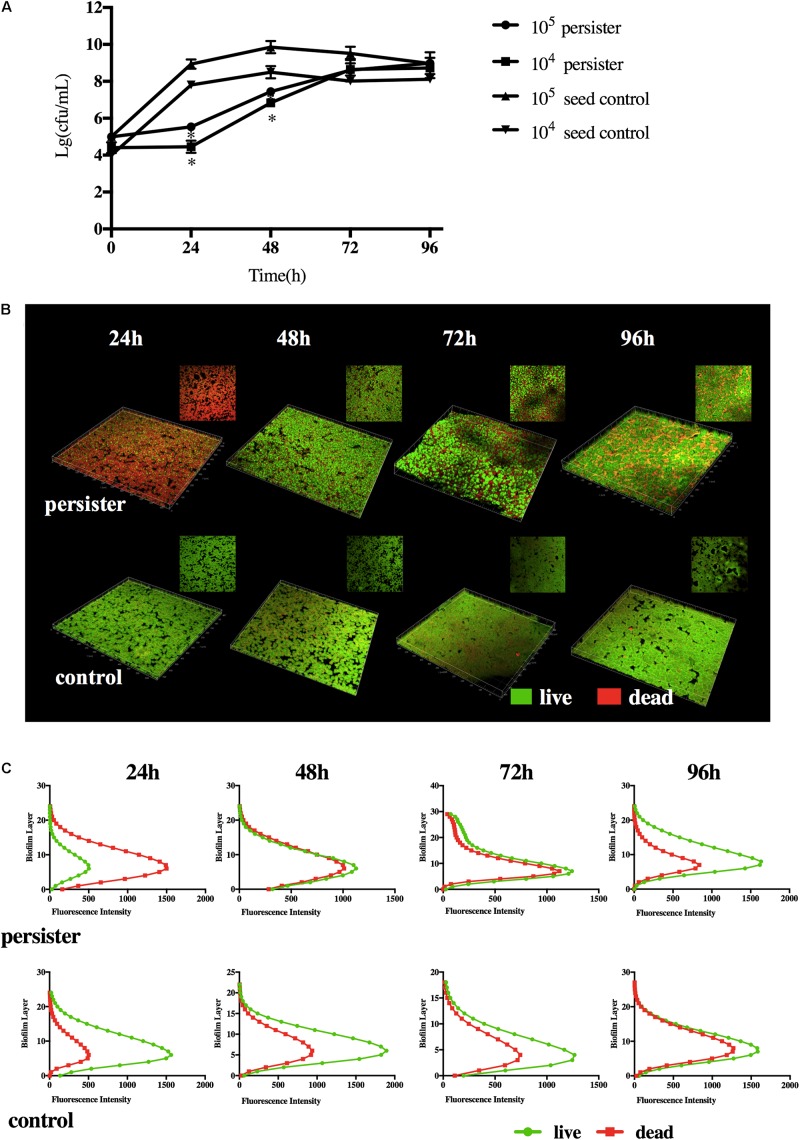
**(A)** Regrowth curve of DMADDM induced *S. mutans* persister and seed control biofilm. Experiments were repeated at least three times. Standard deviation of the mean is shown. Student’s *t*-test was used to compare the data of two groups. Asterisks indicate statistical significance (*p* < 0.05). **(B)** Representative live/dead staining images of DMADDM induced *S. mutans* persister cells and untreated seed control biofilm during recovery periods (live bacteria, stained green; dead bacteria, stained red). **(C)** The fluorescence intensity of live/dead bacteria at each layer of the biofilm during recovery periods.

When the DMADDM was washed with fresh growth medium, *S. mutans* persisters can regrow in the biofilm. Compared with 10^5^ and 10^4^ CFU seed population, persisters remained dormant within the first 24 h, then it turned into the fast-growing period. Persister biofilms did not reach the maximum population until 72 h. They finally formed a stable biofilm at 96 h compared to the seed control groups (Figure 2A). The distribution of persister cells and biofilm structure at different time points was observed by CLSM imaging (Figures 2B,C). Persisters regrown at 24 h displayed only a few viable cells and mostly dead cells (stained red) in the biofilm, and the distribution and structure were similar to the biofilm in persistent stage (Figures 1D, 2B), indicating the lag regrowth of persister cells. The rapid increase of live cells occurred at 48 h in persister biofilm, while the untreated seed biofilm displayed massive live cells (stained green) at first 24 h (Figure 2B). In the persister biofilms, the live cells were significantly lower than the dead cells at 24 h, but reached to the same level at 48–72 h (Figure 2C). When at 96 h, the live cells were significantly higher than the dead cells even higher than the seed control group at this time point (Figure 2C). The distribution and structure of persister biofilm at 96 h were similar with the seed control biofilms at 48 and 72 h (Figure 2C), which was attributed to the long lag phase of persisters (Figure 2A).

### Resumptive Persisters Elevated the Synthesis of Extracellular Polysaccharides

**FIGURE 3:**
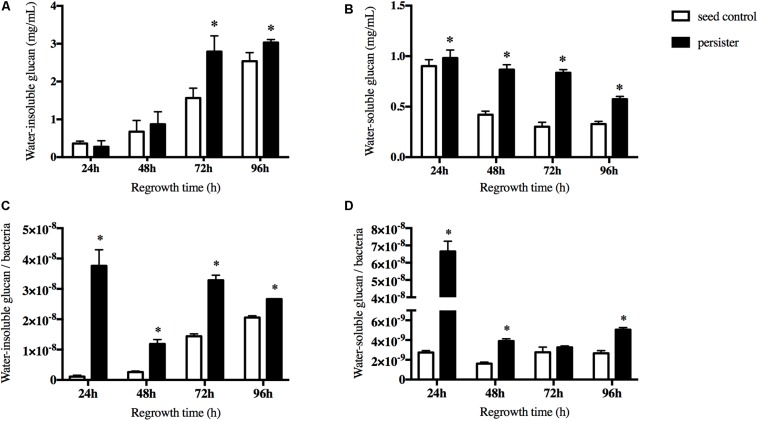
The synthesis of water soluble/insoluble exopolysaccharides (EPS) during recovery periods. **(A)** The water-insoluble glucans of persister and untreated seed control biofilms at indicated times. **(B)** The water-soluble glucans of persister and untreated seed control biofilms at indicated times. **(C)** Average production of water-insoluble glucans within bacteria unit. **(D)** Average production of water-soluble glucans within bacteria unit. Experiments were repeated at least three times. Student’s *t*-test was used to compare the data of two groups. Data are presented as mean ± standard deviation. ^∗^*P* < 0.05.

The extracellular polysaccharides (EPS) is the main cariogenic virulence factor of *S. mutans*. We then measured both the water-soluble and water-insoluble glucans when persisters regrew to a new biofilm. We excluded the pre-accumulation of water-insoluble glucans before the regrowth of persisters in the biofilm. During the first 48 h regrowth stage, there were no significant difference between two groups. After 48 h, the accumulation of water-insoluble glucans in persisters was significantly increased compared to the seed control (Figure 3A). The ability of persisters to produce water-soluble glucans was elevated significantly but gradually reduced over the regrowth stage (Figure 3B). In order to ensure that the elevated ability of EPS synthesis of persisters was not due to disparity of the bacterial load, viable counts were performed. The result showed that both water-insoluble glucan per bacteria and water-soluble glucan per bacteria were increased in persisters (Figures 3C,D), suggesting that persisters can elevate their cariogenic virulence through the increase of EPS production compared to the untreated seed control.

### Resumptive Persisters Gradually Recovered the Acid Production

**FIGURE 4:**
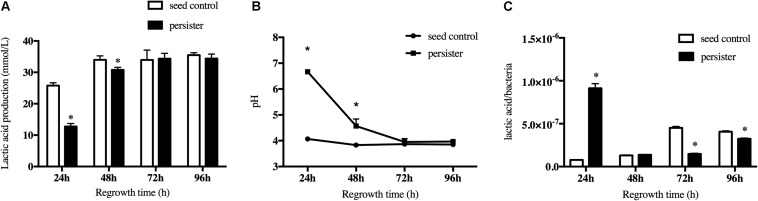
Lactic acid production of persister and untreated seed control biofilms during recovery periods. **(A)** Lactic acid production of 24-, 48-, 72-, and 96-h persister and untreated seed control biofilms. **(B)** The supernatant pH of persister and control biofilms. **(C)** Average production of lactic acid production within bacteria unit. Experiments were repeated at least three times. Student’s *t*-test was used to compare the data of two groups. Data are presented as mean ± standard deviation. ^∗^*P* < 0.05.

Another key cariogenic virulence of *S. mutans* is the acid production. The lactic acid production of persisters decreased in the first regrowth 24 h compared to seeding control due to the long lag phase, but it rapidly increased over time and finally reached the same level as seed control when the biofilm regrew to 72 h (Figure 4A). This was also confirmed by the pH test (Figure 4B). The pH value dropped <4.5 after regrowth to 48 h and kept the same level as the seed control after that. Since there was a difference in bacteria load between persister and seed control biofilm when they regrew to 24–72 h, we calculated the lactic acid produced per bacteria from the groups of persister and seed control. As showed in Figure 4C, the lactic acid/bacteria from the persisters was significantly increased at 24 h, indicating the active fermentation and acid production of persisters, and gradually decreased after 72 and 96 h.

### Quorum-Sensing and VicRK Pathways Were Involved in the Regrowth Persister’s Virulence Variation

**FIGURE 5:**
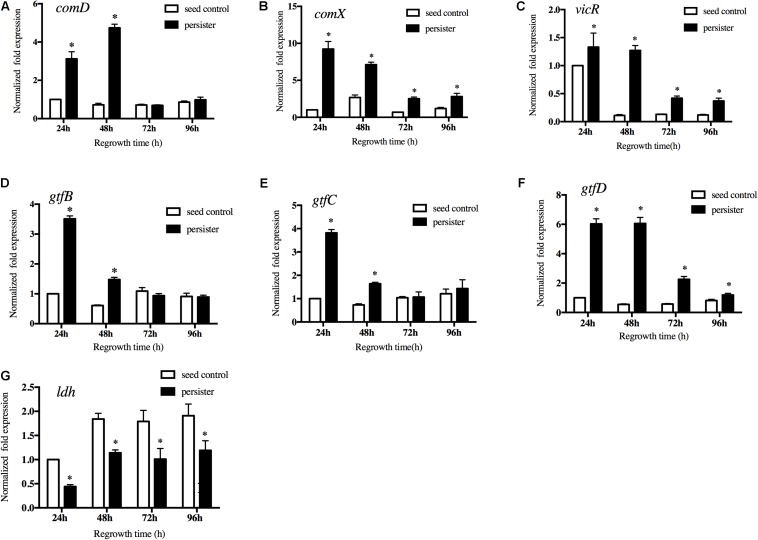
Gene expressions of **(A)** *comD*, **(B)** *comX*, **(C)** *vicR*, **(D)** *gtfB*, **(E)** *gtfC*, **(F)** *gtfD*, and **(G)** *ldh* by real-time quantitative polymerase chain reaction. Experiments were repeated at least three times. Student’s *t*-test was used to compare the data of two groups. Data are presented as mean ± standard deviation. ^∗^*P* < 0.05.

Quorum-sensing system of *S. mutans* played key roles in cell growth and virulence. We then measured the expression of the key genes in quorum-sensing pathway. The results showed that expression of *comD* and *comX* in persisters were significantly up-regulated compared to the seed control (Figures 5A,B). We also measured the expression of *vicR*, a response regulator of the VicRK two-component system that modulates biofilm formation. Persisters significantly enhanced the *vicR* expression compared to the untreated seed biofilm, especially in the early regrowth stage (Figure 5C). We then analyzed the expression of *vicR* regulated downstream genes associated with the EPS and acid production. *gtfB* and *gtfC* in persisters were significantly increased in the first 48 h of regrowth (Figures 5D,E), while *gtfD* in persisters was significantly upregulated from early regrowth stage till 96 h compared to seed control (Figure 5F) consistent with the increased EPS production in persister biofilm (Figure 3). Another virulence gene *ldh* responsible for the lactic acid production was down-regulated in persisters compared to the seed control, and gradually increased during regrowth stage (Figure 5G). The results were in line with the acid production in the persister biofilm (Figure 4).

## Discussion

Persister cells are commonly associated with chronic and recurrent infections. When there is no active antimicrobial agent, the surviving persister subpopulation has the potential to resume a new generation and brings back the initiation of infection. The understanding of persisters’ regrowth can provide new strategy to treat the recurrent infections (Rhen et al., 2003; Lewis, 2010; Fisher et al., 2017).

*Streptococcus mutans* is one of the leading species associated with human dental caries and considered to be the most cariogenic of all the oral streptococci. The present study proved that DMADDM can induce *S. mutans* drug-tolerant persister cells. Increased concentration of DMADDM led to decreased persister level. This would not be the case if all the persisters were pre-existing in the population, as the pre-existing persisters would not be killed by any bactericidal concentration of DMADDM, but keep the same level of persister population. The results suggested that the persister cells in the biofilm were mainly induced by DMADDM rather than pre-existing. Same method by applying different concentrations of antibiotics to *E. coli* also proved the inducible mechanism of persister formation with exposure to fluoroquinolones (Dorr et al., 2009). In this study, we found the distribution of drug-tolerant persister cells in biofilm induced by DMADDM was similar to normal live/dead bacteria distribution in seed control group, indicating the survival of persisters was not due to the lack of penetration of antimicrobial agent. If DMADDM could not permeate the whole layer of biofilm, the distribution of surviving bacteria would appear mainly in the bottom of the biofilm. When the surviving persister cells were re-cultivated, interestingly, we found the resuscitated persister cells formed an actively growing biofilm, especially in the long time of cultivation. The regrown persister biofilm showed more viable cells over dead cells when compared with untreated normal biofilm at 96 h of cultivation. It suggested that persister cells could re-generate a more viable biofilm if the surviving persisters could not be controlled. A remarkable extension of lag time at first 24 h was observed when *S. mutans* persisters resumed growth, which was confirmed by CFU counting and CLSM imaging. The prolonged lag time indicated the late initiation of growth, which could lead persisters to retain unsusceptible to antimicrobial agents. Such survival strategy of persisters was also observed in *S. aureus*, of which the lag time was over 6 h. This fraction of bacteria with a long lag time tolerate antibiotics better than the normal ones (Vulin et al., 2018). Another study reported a lag time of *E. coli* persisters for 14 h before resumption (Balaban et al., 2004). A recent study revealed that the ability of *E. coli* to recruit functional DnaK–ClpB machineries, which facilitate protein disaggregation in an ATP-dependent manner, determined the lag time for bacterial regrowth (Pu et al., 2019). The diverse of lag time may result from the difference of bacterial species and antimicrobial agents. They all emphasized the key role of lag time in persister resuscitation.

Biofilm formation and acid production are two key virulent factors of *S. mutans*. Glucans of *S. mutans* contribute to the establishment of the extracellular polysaccharide matrix, and then promote the accumulation of other oral microorganisms on the tooth surface (Koo et al., 2006). Production of acid is directly involved in the erosion of hard tissue of tooth (Selwitz et al., 2007). We found that the synthesis of water-insoluble glucans and water-soluble glucans was significantly increased when persisters regrowth, which led to the increased accumulation of EPS compared to untreated biofilms. The *gtfB* and *gtfC* genes are thought to encode enzymes that produce mostly water-insoluble glucans, while *gtfD* encodes an enzyme that synthesizes water-soluble glucans (Koo et al., 2006). In our study, *S. mutans* persisters significantly upregulated the expression of *gtf* genes, especially in the lag time, whereas the glucan production was lower than the control seeding cells. This could be explained by the disparity of bacterial load. By taking the viable cells in count, we found the production of glucan per bacteria was significantly increased in persisters (Figures 3C,D). When the difference of bacterial load was excluded between regrowth persisters untreated seeding group at regrowth time 72 and 96 h, the accumulation of glucans significantly increased in regrowth persisters, suggesting that persisters can elevate their cariogenic virulence through the increase of extracellular polysaccharide matrix production compared to the untreated seed control. Interestingly, the acid production of persisters decreased in the first 48 h, but the ability of individual cell was enhanced. The loss of acid production in persisters could possibly due to a less amount of bacterial population as they remained in the lag phase at 24 h. The elevated virulence of regrown persisters indicated that *S. mutans* persisters could regenerate a more cariogenic biofilm, and persisters were metabolically active although they did not increase the cell number in the prolonged lag time.

The water-soluble glucans, as a rich carbon source, may play an important role depending on the state of persister biofilms. Our previous study proved that in the dormant state, the addition of glucose significantly reduced the number of persister cells (Jiang et al., 2017). It indicated that the addition of carbon sources can reverse the dormancy of the bacteria to resuscitation. In the resuscitation state, the regrowth persister themselves also synthesized increased water-soluble glucans, and *gtfD* was significantly expressed. These glucans not only served as abundant carbon resource for the growth of their own population, but also improved the population survival rate at the population level. These results indicated that metabolic-related pathways may be a potential pathway for resuscitation of *S. mutans* persisters.

Notably, the lag–regrowth phase may be the critical target for eradicating persisters by blocking the metabolic pathways since *S. mutans* persisters are metabolically active in “lag time.” Quorum-sensing system is commonly thought to participate in pathogenicity and metabolic adjustment in *S. mutans* (Suntharalingam and Cvitkovitch, 2005; Li et al., 2008). CSP-ComDE circuit is a well-conserved signaling peptide-mediated quorum-sensing system. *comD* gene encodes a membrane-bound histidine kinase (HK) sensor protein. *comX* is a homolog of an alternate sigma factor that directs transcription of RNA polymerase to a number of competence-related genes as part of the CSP-induced signal cascade (Son et al., 2015). In our study, *comD* and *comX* were significantly up regulated in *S. mutans* persisters during the lag phase and the first 48 h of regrowth, which were in line with the increased synthesis of EPS. We also analyzed the expression of *vicR* gene, a response regulator of the VicRK two-component system that modulates biofilm formation, and its regulated virulent genes *gtfs* responsible for the synthesis of EPS and lactic acid. In our study, *S. mutans* persisters significantly upregulated the expression of *vicR* and *gtf* genes in line with the production of extracellular glucans. The *ldh* gene responsible for the lactic acid biosynthesis was down-regulated in the persisters, which is consistent with the acid production. In our previous work, we have monitored the expression of several key glycolysis- and citrate cycle-related genes in *S. mutans* persister cells, including *adhE*, *ldh*, *and pdhA/B/C/D.* The expression levels of these genes were all significantly downregulated in persistent cells compared to the control group, suggesting the low metabolic level of *S. mutans* persisters (Jiang et al., 2017). Contrarily, in this study we observed the *S. mutans* persisters were metabolically active in “lag time” when they reverse from dormancy to regrowth in terms of quorum-sensing and two component system VicRK pathways. This result revealed the conversion of metabolic activity from dormancy of persisters to regrowth. Interestingly, the related genes were notably over expressed in lag time. It implied the key role of quorum-sensing and two component system VicRK pathways in regulating the metabolic activity in preparation for persister resuscitation in lag phase. This hypothesis was in agreement with a recent research that reported the increased protein level in prior to the regrowth of persisters (Pu et al., 2019). They found that for cells to leave the dormant state and resuscitate, clearance of intracellular protein aggresome was required. In *E. coli*, DnaK and ClpB are responsible for dissolving protein aggregates. The protein level of DnaK was initially low when persister cells were dormant, but cellular DnaK levels increased substantially, colocalizing with protein aggregates prior to the persister resuscitation. After that, these protein aggregates dissolved, and cell regrowth was initiated. As is in our study, quorum-sensing and two component system VicRK pathways genes were up-regulated in lag time prior to the fast regrowth of *S. mutans* persisters. Our results revealed the dynamic expressions and key roles of quorum-sensing and VicRK pathway genes in the regrowth of *S. mutans* persisters for the first time. However, more elaborated regulation pathways need to be further clarified. We intend to explore these questions in our future studies. It was also suggested that blocking these two pathways may inhibit the *S. mutans* presisters regrowth and reduce the recurrence of new caries.

## Conclusion

Dimethylaminododecyl methacrylate induced *S. mutans* persister cells showed an increase of virulence when they regrew. This alarmed the elevated cariogenicity of persisters and highlighted the critical requirement for the drug-tolerance evaluation when developing new oral antimicrobial agents. The prolonged lag phase of *S. mutans* persisters was about 24 h and could be a new window for the eradication of the persistent cells. The involvement of quorum-sensing and VicRK pathways in the resumption process may provide potential idea for innovative drug designs and strategies to overcome bacterial persistence.

## Data Availability Statement

All datasets generated for this study are included in the article/Supplementary Material.

## Author Contributions

JL, LC, HX, XZ, and BR conceived and designed the study. JL, YH, and YJ performed the experiments and collected the data. JL, LC, C-HC, XP, ML, and BR analyzed and interpreted the data. JL drafted the manuscript. LC and BR critically revised the manuscript. JL, LC, YH, YJ, C-HC, XP, ML, HX, XZ, and BR approved the final version of the manuscript.

## Conflict of Interest

The authors declare that the research was conducted in the absence of any commercial or financial relationships that could be construed as a potential conflict of interest.

## References

[B1] Balaban N. Q. Gerdes K. Lewis K. McKinney J. D. (2013). A problem of persistence: still more questions than answers? *Nat. Rev. Microbiol.* 11 587–591. 10.1038/nrmicro3076 24020075

[B2] Balaban N. Q. Merrin J. Chait R. Kowalik L. Leibler S. (2004). Bacterial persistence as a phenotypic switch. *Science* 305 1622–1625. 10.1126/science.1099390 15308767

[B3] Beyth N. Yudovin-Farber I. Bahir R. Domb A. J. Weiss E. I. (2006). Antibacterial activity of dental composites containing quaternary ammonium polyethylenimine nanoparticles against *Streptococcus mutans*. *Biomaterials* 27 3995–4002. 10.1016/j.biomaterials.2006.03.003 16564083

[B4] Brauner A. Fridman O. Gefen O. Balaban N. Q. (2016). Distinguishing between resistance, tolerance and persistence to antibiotic treatment. *Nat. Rev. Microbiol.* 14 320–330. 10.1038/nrmicro.2016.34 27080241

[B5] Cheng L. Weir M. D. Zhang K. Arola D. D. Zhou X. Xu H. H. (2013). Dental primer and adhesive containing a new antibacterial quaternary ammonium monomer dimethylaminododecyl methacrylate. *J. Dent.* 41 345–355. 10.1016/j.jdent.2013.01.004 23353068 PMC3631010

[B6] Cheng L. Zhang K. Zhang N. Melo M. A. S. Weir M. D. Zhou X. D. (2017). Developing a new generation of antimicrobial and bioactive dental resins. *J. Dent. Res.* 96 855–863. 10.1177/0022034517709739 28530844 PMC5502962

[B7] Cohen N. R. Lobritz M. A. Collins J. J. (2013). Microbial persistence and the road to drug resistance. *Cell Host Microbe* 13 632–642. 10.1016/j.chom.2013.05.009 23768488 PMC3695397

[B8] Dorr T. Lewis K. Vulic M. (2009). SOS response induces persistence to fluoroquinolones in *Escherichia coli*. *PLoS Genet* 5:e1000760. 10.1371/journal.pgen.1000760 20011100 PMC2780357

[B9] Fisher R. A. Gollan B. Helaine S. (2017). Persistent bacterial infections and persister cells. *Nat. Rev. Microbiol.* 15 453–464. 10.1038/nrmicro.2017.42 28529326

[B10] Gefen O. Balaban N. Q. (2009). The importance of being persistent: heterogeneity of bacterial populations under antibiotic stress. *FEMS Microbiol. Rev.* 33 704–717. 10.1111/j.1574-6976.2008.00156.x 19207742

[B11] Helaine S. Kugelberg E. (2014). Bacterial persisters: formation, eradication, and experimental systems. *Trends Microbiol.* 22 417–424. 10.1016/j.tim.2014.03.008 24768561

[B12] Holmstrup P. Klausen B. (2016). The growing problem of antimicrobial resistance. *Oral Dis.* 24 291–295. 10.1111/odi.12610 27860048

[B13] Horne D. Tomasz A. (1977). Tolerant response of Streptococcus sanguis to beta-lactams and other cell wall inhibitors. *Antimicrob. Agents Chemother.* 11 888–896. 10.1128/aac.11.5.888 879739 PMC352092

[B14] Imazato S. (2009). Bio-active restorative materials with antibacterial effects: new dimension of innovation in restorative dentistry. *Dent. Mater. J.* 28 11–19. 10.4012/dmj.28.11 19280964

[B15] Jiang Y.-L. Qiu W. Zhou X.-D. Li H. Lu J.-Z. Xu H. H. K. (2017). Quaternary ammonium-induced multidrug tolerant *Streptococcus mutans* persisters elevate cariogenic virulence in vitro. *Int. J. Oral Sci.* 9:e7 10.1038/ijos.2017.46 PMC5750454 32987970

[B16] Koo H. Hayacibara M. F. Schobel B. D. Cury J. A. Rosalen P. L. Park Y. K. (2003). Inhibition of *Streptococcus mutans* biofilm accumulation and polysaccharide production by apigenin and tt-farnesol. *J. Antimicrob. Chemother.* 52 782–789. 10.1093/jac/dkg449 14563892

[B17] Koo H. Seils J. Abranches J. Burne R. A. Bowen W. H. Quivey R. G. Jr. (2006). Influence of apigenin on gtf gene expression in *Streptococcus mutans* UA159. *Antimicrob. Agents Chemother.* 50 542–546. 10.1128/AAC.50.2.542-546.2006 16436708 PMC1366919

[B18] Lafleur M. D. Qi Q. Lewis K. (2010). Patients with long-term oral carriage harbor high-persister mutants of *Candida albicans*. *Antimicrob. Agents Chemother.* 54 39–44. 10.1128/AAC.00860-09 19841146 PMC2798516

[B19] Leung V. Ajdic D. Koyanagi S. Levesque C. M. (2015). The formation of *Streptococcus mutans* persisters induced by the quorum-sensing peptide pheromone is affected by the LexA regulator. *J. Bacteriol.* 197 1083–1094. 10.1128/JB.02496-14 25583974 PMC4336345

[B20] Leung V. Levesque C. M. (2012). A stress-inducible quorum-sensing peptide mediates the formation of persister cells with noninherited multidrug tolerance. *J. Bacteriol.* 194 2265–2274. 10.1128/JB.06707-11 22366415 PMC3347057

[B21] Lewis K. (2007). Persister cells, dormancy and infectious disease. *Nat. Rev. Microbiol.* 5 48–56. 10.1038/nrmicro1557 17143318

[B22] Lewis K. (2010). Persister cells. *Annu. Rev. Microbiol.* 64 357–372. 10.1146/annurev.micro.112408.134306 20528688

[B23] Lewis K. Shan Y. (2016). Persister awakening. *Mol. Cell* 63 3–4. 10.1016/j.molcel.2016.06.025 27392143

[B24] Li Y. H. Tian X. L. Layton G. Norgaard C. Sisson G. (2008). Additive attenuation of virulence and cariogenic potential of *Streptococcus mutans* by simultaneous inactivation of the ComCDE quorum-sensing system and HK/RR11 two-component regulatory system. *Microbiology* 154 (Pt 11), 3256–3265. 10.1099/mic.0.2008/019455-0 18957580

[B25] Liang J. Li M. Ren B. Wu T. Xu H. H. K. Liu Y. (2018). The anti-caries effects of dental adhesive resin influenced by the position of functional groups in quaternary ammonium monomers. *Dent. Mater.* 34 400–411. 10.1016/j.dental.2017.11.021 29269159

[B26] McKeegan K. S. Borges-Walmsley M. I. Walmsley A. R. (2002). Microbial and viral drug resistance mechanisms. *Trends Microbiol.* 10 (10 Suppl.), S8–S14. 12377562 10.1016/s0966-842x(02)02429-0

[B27] Mei M. L. Li Q. L. Chu C. H. Lo E. C. Samaranayake L. P. (2013). Antibacterial effects of silver diamine fluoride on multi-species cariogenic biofilm on caries. *Ann. Clin. Microbiol. Antimicrob.* 12:4. 10.1186/1476-0711-12-4 23442825 PMC3599989

[B28] Mina E. G. Marques C. N. (2016). Interaction of Staphylococcus aureus persister cells with the host when in a persister state and following awakening. *Sci. Rep.* 6:31342. 10.1038/srep31342 27506163 PMC4979210

[B29] Monack D. M. Mueller A. Falkow S. (2004). Persistent bacterial infections: the interface of the pathogen and the host immune system. *Nat. Rev. Microbiol.* 2 747–765. 10.1038/nrmicro955 15372085

[B30] Page R. Peti W. (2016). Toxin-antitoxin systems in bacterial growth arrest and persistence. *Nat. Chem. Biol.* 12 208–214. 10.1038/nchembio.2044 26991085

[B31] Palmer G. H. Bankhead T. Lukehart S. A. (2009). ‘Nothing is permanent but change’- antigenic variation in persistent bacterial pathogens. *Cell Microbiol.* 11 1697–1705. 10.1111/j.1462-5822.2009.01366.x 19709057 PMC3354987

[B32] Peng X. Michalek S. Wu H. (2016). Effects of diadenylate cyclase deficiency on synthesis of extracellular polysaccharide matrix of *Streptococcus mutans* revisit. *Environ. Microbiol.* 18 3612–3619. 10.1111/1462-2920.13440 27376962

[B33] Pu Y. Li Y. Jin X. Tian T. Ma Q. Zhao Z. (2019). ATP-Dependent Dynamic Protein Aggregation Regulates Bacterial Dormancy Depth Critical for Antibiotic Tolerance. *Mol. Cell* 73 143.e4–156.e4. 10.1016/j.molcel.2018.10.022 30472191

[B34] Pu Y. Zhao Z. Li Y. Zou J. Ma Q. Zhao Y. (2016). enhanced efflux activity facilitates drug tolerance in dormant bacterial cells. *Mol. Cell* 62 284–294. 10.1016/j.molcel.2016.03.035 27105118 PMC4850422

[B35] Rhen M. Eriksson S. Clements M. Bergström S. Normark S. J. (2003). The basis of persistent bacterial infections. *Trends Microbiol.* 11 80–86. 10.1016/S0966-842X(02)00038-0 12598130

[B36] Selwitz R. H. Ismail A. I. Pitts N. B. (2007). Dental caries. *Lancet* 369 51–59. 10.1016/s0140-6736(07)60031-2 17208642

[B37] Son M. Shields R. C. Ahn S. J. Burne R. A. Hagen S. J. (2015). Bidirectional signaling in the competence regulatory pathway of *Streptococcus mutans*. *FEMS Microbiol. Lett.* 362:fnv159. 10.1093/femsle/fnv159 26363019 PMC4809993

[B38] Suntharalingam P. Cvitkovitch D. G. (2005). Quorum sensing in streptococcal biofilm formation. *Trends Microbiol.* 13 3–6. 10.1016/j.tim.2004.11.009 15639624

[B39] van Loveren C. Buijs J. F. ten Cate J. M. (2000). The effect of triclosan toothpaste on enamel demineralization in a bacterial demineralization model. *J. Antimicrob. Chemother.* 45 153–158. 10.1093/jac/45.2.153 10660496

[B40] Vulin C. Leimer N. Huemer M. Ackermann M. Zinkernagel A. S. (2018). Prolonged bacterial lag time results in small colony variants that represent a sub-population of persisters. *Nat Commun* 9:4074. 10.1038/s41467-018-06527-0 30287875 PMC6172231

[B41] Wang S. P. Ge Y. Zhou X. D. Xu H. H. Weir M. D. Zhang K. K. (2016). Effect of anti-biofilm glass-ionomer cement on *Streptococcus mutans* biofilms. *Int. J. Oral Sci.* 8 76–83. 10.1038/ijos.2015.55 27357319 PMC4932770

[B42] Zhang K. Ren B. Zhou X. Xu H. H. Chen Y. Han Q. (2016). Effect of antimicrobial denture base resin on multi-species biofilm formation. *Int. J. Mol. Sci.* 17:1033. 10.3390/ijms17071033 27367683 PMC4964409

[B43] Zhang K. Wang S. Zhou X. Xu H. H. Weir M. D. Ge Y. (2015). Effect of antibacterial dental adhesive on multispecies biofilms formation. *J. Dent. Res.* 94 622–629. 10.1177/0022034515571416 25715378 PMC4485219

