# Supplementary material for: Resumptive Streptococcus mutans Persisters Induced From Dimethylaminododecyl Methacrylate Elevated the Cariogenic Virulence by Up-Regulating the Quorum-Sensing and VicRK Pathway Genes
Source: Front Microbiol. 2020 Jan 21;10:3102. doi: 10.3389/fmicb.2019.03102 (PMC6985435; doi:10.3389/fmicb.2019.03102)
Supplement: Supplementary file 1 [file Data_Sheet_1.docx]

Supplementary Material

# Supplementary Figures and Tables

## Supplementary Figure 1

## Supplementary Figure 1. The schematic illustration of experiment route of persister inducing and regrowth.

## Supplementary Tables

Supplementary Table 1. Name and description of genes uesd in this study

| Gene name | Gene description |
| --- | --- |
| *gtf B* | glucosyltransferase-I |
| *gtf C* | Glucosyltransferase-SI |
| *gtf D* | Glucosyltransferase-S |
| *ldh* | L-lactate dehydrogenase |
| *vicR* | response regulator VicR |
| *comD*  *comX* | histidine kinase of the competence regulon,ComD  competence protein ComX |

Supplementary Table 2. Primers of quantitative real-time PCR

| **Primer** | **Sequence (F and R)** |
| --- | --- |
| **16S rRNA** | 5’-AGCGTTGTCCGGATTTATTG-3’  5’-CTACGCATTTCACCGCTACA-3’ |
| ***gtf B*** | 5’-CACTATCGGCGGTTACGAAT-3’  5’-CAATTTGGAGCAAGTCAGCA-3’ |
| ***gtf C*** | 5’-GATGCTGCAAACTTCGAACA-3’  5’-TATTGACGCTGCGTTTCTTG-3’ |
| ***gtf D***  ***ldh***  ***vicR***  ***comD***  ***comX*** | 5’-TTGACGGTGTTCGTGTTGAT-3’  5’-AAAGCGATAGGCGCAGTTTA-3’  5’-CCTCGTTGCTGCTAACCCAG-3’  5’-TGCTTGACGGAAACGAGCAG-3’  5’-GCGTCATCACCTGACCTGTG-3’  5’-CCGCAGTGGCTGAGGAAAA-3’  5’-AGCGCTATTCCTGCAAACTCG-3’  5’-TATGGTCTGCTGCCTGTTGC-3’  5’-CTACCAAGCGCTCAAACAGC-3’  5’-CACTGTTTGTCAAGTGGCGG-3’ |

**Supplementary Table 3. The minimum inhibitory concentration (MIC) of bacteria**

| **Bacteria Strains** | **UA159** | **Persisters** | |
| --- | --- | --- | --- |
|  |  | **10-fold MIC Induced** | **12-fold MIC Induced** |
| MIC（μg/ml) | 9.76 | 9.76(cycle 1) | 9.76(cycle 1) |
|  |  | 9.76(cycle 2) | 9.76(cycle 2) |
|  |  | 9.76(cycle 3) | 9.76(cycle 3) |
